# Pathologic complete response prediction in breast cancer lesion segmentation and neoadjuvant therapy

**DOI:** 10.3389/fmed.2023.1188207

**Published:** 2023-12-04

**Authors:** Yue Liu, Zhihong Chen, Junhao Chen, Zhenwei Shi, Gang Fang

**Affiliations:** ^1^Institute of Computing Science and Technology, Guangzhou University, Guangzhou, China; ^2^School of Information Engineering, Jiangxi College of Applied Technology, Ganzhou, China; ^3^Guangdong Provincial Key Laboratory of Artificial Intelligence in Medical Image Analysis and Application, Guangdong Provincial People's Hospital, Guangdong Academy of Medical Sciences, Guangzhou, China; ^4^Department of Radiology, Guangdong Provincial People’s Hospital, Guangdong Academy of Medical Sciences, Guangzhou, China; ^5^Guangdong Cardiovascular Institute, Guangdong Provincial People's Hospital, Guangdong Academy of Medical Sciences, Guangzhou, China

**Keywords:** PCR, DCE-MRI, neoadjuvant chemotherapy, multi-modal fusion model, breast cancer, lesion segmentation

## Abstract

**Objectives:**

Predicting whether axillary lymph nodes could achieve pathologic Complete Response (pCR) after breast cancer patients receive neoadjuvant chemotherapy helps make a quick follow-up treatment plan. This paper presents a novel method to achieve this prediction with the most effective medical imaging method, Dynamic Contrast-enhanced Magnetic Resonance Imaging (DCE-MRI).

**Methods:**

In order to get an accurate prediction, we first proposed a two-step lesion segmentation method to extract the breast cancer lesion region from DCE-MRI images. With the segmented breast cancer lesion region, we then used a multi-modal fusion model to predict the probability of axillary lymph nodes achieving pCR.

**Results:**

We collected 361 breast cancer samples from two hospitals to train and test the proposed segmentation model and the multi-modal fusion model. Both segmentation and prediction models obtained high accuracy.

**Conclusion:**

The results show that our method is effective in both the segmentation task and the pCR prediction task. It suggests that the presented methods, especially the multi-modal fusion model, can be used for the prediction of treatment response in breast cancer, given data from noninvasive methods only.

## 1 Introduction

The incidence of breast cancer has been increasing in recent years, and breast cancer is one of the most common malignant cancers in women. In 2023, it is estimated that there will be 297,790 new cases of invasive breast cancer diagnosed and 43,170 women will die from breast cancer in the U.S. (1). At present, neoadjuvant chemotherapy (NAC) plays an important role in breast cancer treatment, and research (2–5) shows that whether axillary lymph nodes achieve pathologic Complete Response (pCR) is an important prognostic predictor for breast cancer patients who receive NAC, and that pCR indicates a lower risk of local recurrence and a better long-term prognosis for patients. Therefore, it is of great importance if we can predict whether axillary lymph nodes will achieve pCR after patients receive NAC; this helps make a follow-up treatment plan and improve patients’ prognosis.

As a non-invasive method, imaging examination plays an important role in the clinical diagnosis and treatment of cancer. Specifically, in the evaluation of treatment response to NAC in breast cancer, Magnetic Resonance Imaging (MRI) is the most commonly used imaging evaluation method in clinical practice (6). However, based on radiologists’ subjective evaluation of imaging features, MRI shows high sensitivities (83–92%) and intermediate specificities (47–63%) in preoperative diagnosis of axillary lymph nodes achieving pCR after NAC (7). Nevertheless, recently, artificial intelligence has shown great promise in analyzing medical images, helping to identify image information beyond the ability of the naked eye, and providing objective quantitative evaluation to support clinical decision-making (6). Specifically, deep neural networks, especially convolutional networks, attract great attention in the field of medical imaging analysis (8). Thus, the objective of this paper is to utilize deep neural networks to process MRI images of breast cancer in order to predict whether axillary lymph node metastasis in breast cancer could achieve pCR after patients receive NAC.

**Figure 1:**
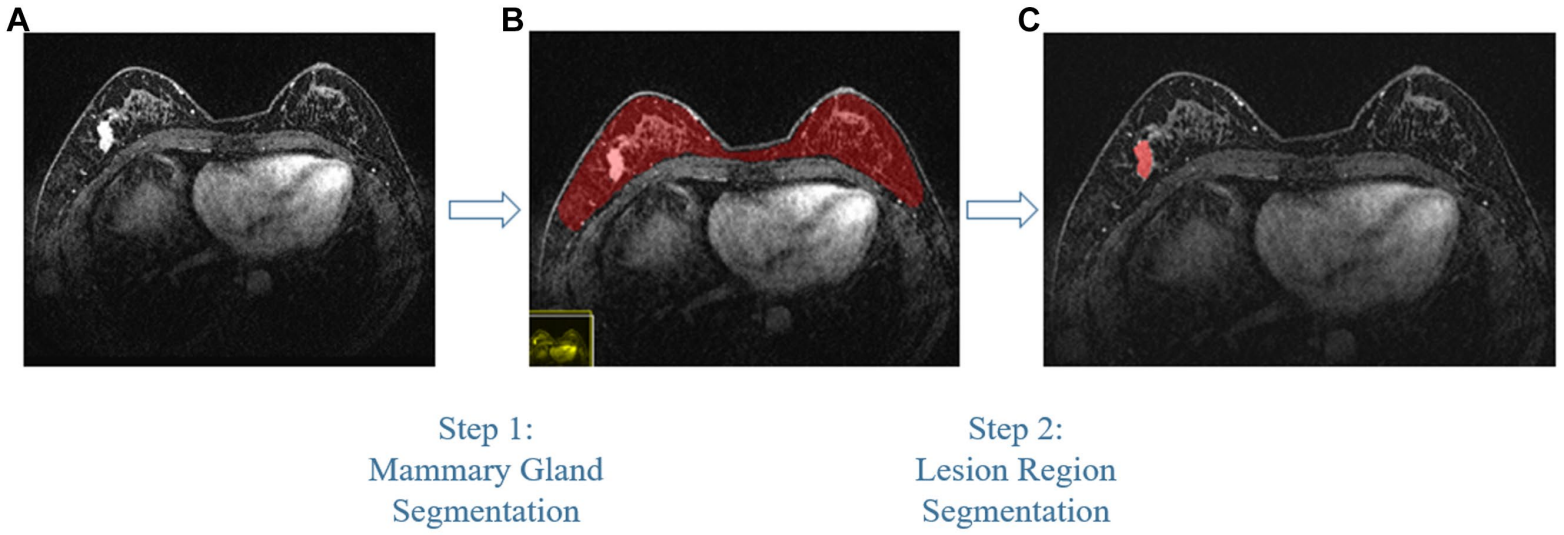
The proposed two-step breast cancer lesion segmentation method. **(A)** is a DCE-MRI image sample for breast cancer diagnosis, which contains irrelevant regions, such as the heart; **(B)** shows the segmented mammary gland region in red; **(C)** shows the breast cancer lesion region in red.

Compared with other types of medical imaging technologies, such as Computed Tomography (CT) and Positron Emission Tomography (PET), MRI provides better imaging capability for soft tissues and is widely adopted in breast cancer diagnosis and treatment. In our work, we use Dynamic Contrast-enhanced Magnetic Resonance Imaging (DCE-MRI), which provides a high-quality image for soft tissues with better quality of blood flow around the lesion, which facilitates higher accuracy and earlier detection in breast cancer diagnosis. Despite the above, due to the nature of medical imaging technology, a common DCE-MRI image for breast cancer diagnosis (Figure 1A), contains a large amount of redundant information, so we need to extract only the lesion region of interest for further processing in order to achieve better performance. Image segmentation is widely used in medical imaging analysis, where pixels from specific regions are segmented from the background. With the prevalence of deep learning, models such as the Fully Convolutional Network (FCN) (9) and UNet (10) are applied in medical image segmentation and achieve great performance. It is also proven that neural networks are effective and efficient in breast tumor segmentation tasks (11). In our work, considering the fact that breast cancer lesions are close to the chest wall and vary in size and distribution, we propose a two-step lesion segmentation method using nnUNet (12) as shown in Figure 1: (1) segmentation of the mammary gland region; (2) segmentation of the breast cancer lesion region within the mammary gland region. When training medical image segmentation models, transferability should be taken into consideration because DCE-MRI images collected from different centers may vary in resolution, scanner used, protocol, and image quality. Hence, we apply a histogram matching method (13) to augment the training samples in order to improve the model transferability.

Usually, after acquiring the model representation of breast cancer lesions, we can directly train a neural network to predict the probability of pCR after NAC. However, as pointed out by Ramos-Vara (14), immunohistochemical detection can greatly help in the diagnosis of breast cancer, invasion and metastasis of tumors, and prognosis of breast cancer, so together with MRI data, we coordinate four common types of molecular typing data in breast cancer treatment to construct a multi-modal fusion model to predict whether axillary lymph nodes could achieve pCR. Our experiments show that the proposed multi-modal fusion model outperforms the predictive model with only MRI data.

In order to train and evaluate the proposed two-step lesion segmentation method and the multi-model fusion model, we collected 361 breast cancer samples from two hospitals: 246 samples from Guangdong Provincial People’s Hospital using the Philips Achieva 1.5 T MRI system, and 115 samples from Henan Renmin Hospital using the Discovery MR750 3.0 T MRI system. Each sample comes with DCE-MRI imaging and molecular typing data, and each DCE-MRI image is labeled and verified by radiologists with more than 5 years of breast cancer experience.

In this paper, we make the following three contributions: First, we propose a two-step lesion segmentation method to extract breast cancer lesion regions from DCE-MRI images. In the model training, considering the different sources of DCE-MRI images, we apply a simple histogram matching method to improve the model transferability. Second, we propose a multi-modal (i.e., segmented DCE-MRI image and molecular typing data) fusion model to predict the probability of axillary lymph nodes achieving pCR after patients receive neoadjuvant therapy. Finally, we evaluate our model through extensive ablation studies and experiments on a collected breast cancer dataset, and we show the promising performance of the proposed method.

The studies involving human participants were reviewed and approved by the Ethics Board of Guangdong Provincial People’s Hospital and the Ethics Board of Henan Renmin Hospital. Written informed consent to participate in this study was provided by the participants.

## 2 Related works

### 2.1 Convolutional neural network

Convolutional neural networks (CNN) have achieved great success in medical imaging analysis. CNN was first introduced to process medical images by Lo et al. (15), and with the rapid development of CNN (16, 17), it has been considered one of the most effective methods to process medical images. ResNet (18), as one of the most classic CNNs, is widely adopted in all kinds of neural networks; with 152 layers of networks, it outperformed other models in the ImageNet Large Scale Visual Recognition Challenge (ILSVRC) 2015 by a large margin. Compared with AlexNet (16) and VGGNet (19), ResNet achieves smaller training errors and better testing accuracy, so in our proposed models, we chose ResNet as the backbone network.

### 2.2 Medical image segmentation

With the rapid development and popularization of medical imaging devices and technologies, including MRI, CT, PET, etc., the amount of medical images produced by these devices is increasing; it is reported that medical images account for one-fifth of all images generated worldwide. Thus, it is urgent to process medical images effectively, and medical image segmentation is the first important step in image analysis. Among all medical imaging technologies, MRI is the most widely adopted one. With MRI, professionals can vary the image contrast to show different image intensities to reflect the difference between soft tissue, parenchyma, and fluid (20, 21).

With the assistance of CNN, various medical image segmentation methods have been developed. In 2015, FCN (9) was proposed to implement pixel-level classification to solve the semantic segmentation problem; it accepts images of arbitrary sizes. FCN was one of the first deep learning techniques that were applied to medical image processing, but the segmentation performance is not satisfactory. Based on FCN, Olaf et al. introduced U-Net (10) for cell image segmentation; its surprising performance soon made it a standard backbone network. Later, 3D U-Net (22), V-Net (23), Res-UNet (24), and other variants of U-Net (25–30) were proposed. Aside from FCN and U-Net, Recurrent Neural Networks (RNNs) are also utilized for medical image segmentation (31, 32).

### 2.3 Pathologic complete response prediction

Over the last decade, many methods have been developed in academia to predict pCR, including radiomics, machine learning, and deep learning. In radiomics, pre-designed features are extracted to build a predictive model, but these pre-designed features are complex (33). Traditional machine learning methods such as SVM and AdaBoost also need well-designed features for prediction. In (34), 13,950 imaging features are extracted from CT and MRI data for machine learning. Compared with traditional radiomics and machine learning methods, the predictive model based on deep learning provides an end-to-end training and inferring method that can be directly applied to medical images (35). In our work, we use MRI images of breast cancer lesion regions, along with four types of molecular typing data commonly used in breast cancer treatment, to construct a multi-modal fusion model to predict whether or not the patient can achieve pCR.

## 3 Methods

In this section, we introduce the proposed method of processing MRI images of breast cancer with neural networks in order to predict whether axillary lymph node metastasis in breast cancer could achieve pCR after patients receive neoadjuvant therapy. We have divided this section into two parts: the first part gives details on how we extract the breast cancer lesion region from a common DCE-MRI breast cancer image; the second part introduces the multi-modal fusion model for pCR prediction.

### 3.1 Breast cancer lesion segmentation

**Figure 2:**
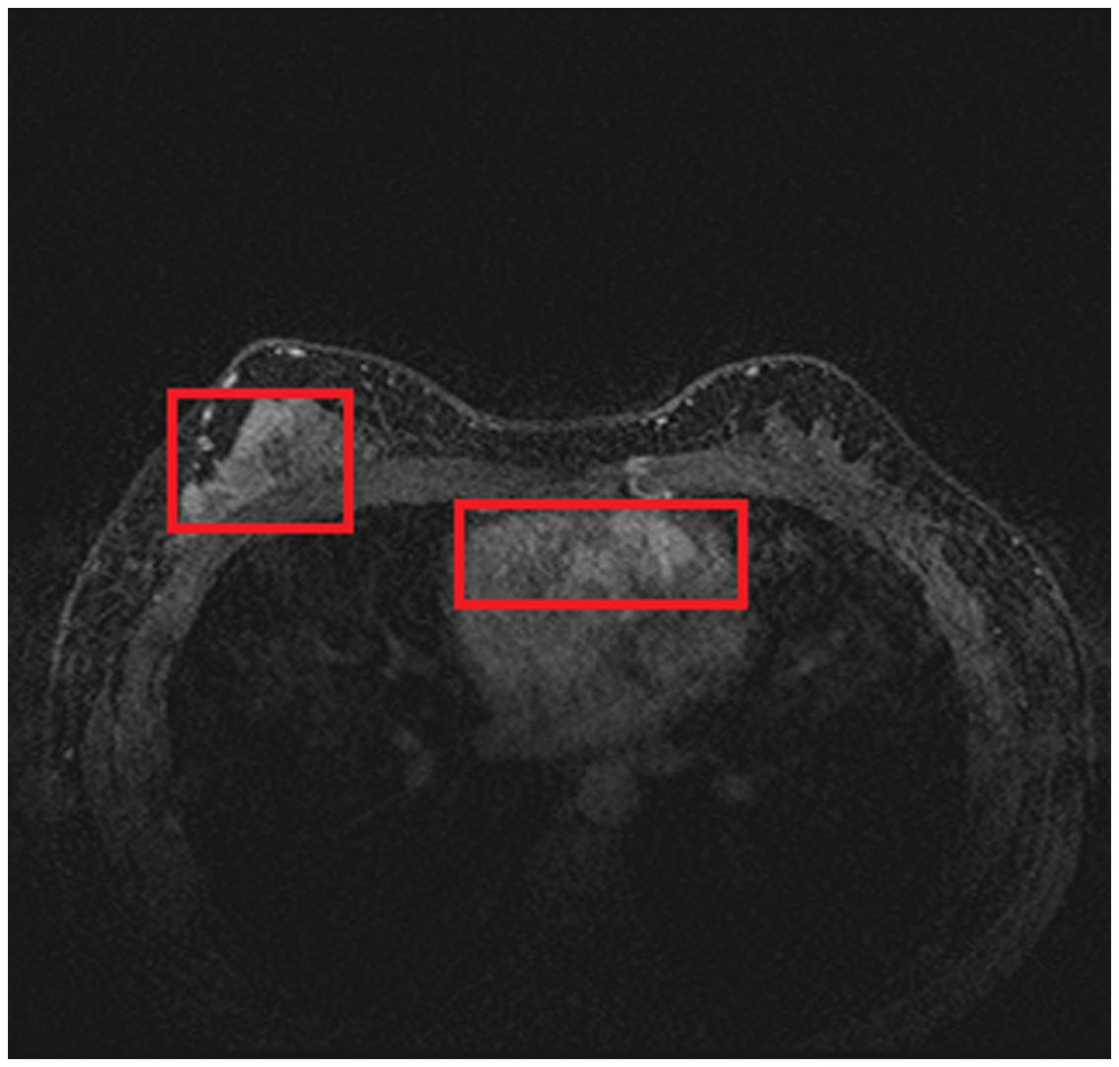
Two red rectangles show similar imaging intensity in the breast and heart regions.

As introduced in Section 1, and referring to Figure 1A, a common DCE-MRI image for breast cancer diagnosis contains a large amount of information that is irrelevant, so we needed to extract only the concerned lesion region for later processing in order to achieve better performance. Another notable reason for breast cancer lesion segmentation is that there is similar imaging intensity in the heart region in DCE-MRI images, as shown in Figure 2, so it is preferable to remove the heart region in order to reduce the probability of false positives. In the following, we elaborated on the proposed two-step lesion segmentation method to extract breast cancer lesion regions based on nnUNet and introduced a simple histogram matching method to augment the training samples in order to improve the model transferability between different centers.

#### 3.1.1 Backbone network for image segmentation

As the DCE-MRI data collected for training is three-dimensional, we chose nnUNet (12) as the backbone network for the breast cancer lesion segmentation task. We specified the nnUNet as follows:

##### 3.1.1.1 Pre-processing

Re-sampling and normalization were implemented at this stage. As the spatial resolution of each MRI image varied, which means one pixel of the image may represent a different size of physical space, the MRI image needs to be re-sampled according to the median of the spatial resolution of all data. Z-score normalization was done independently for each patient’s imaging data.

Data augmentation was also implemented. Augmentation techniques include random rotation, random scaling, random elastic transformation, gamma correction, and inversion.

##### 3.1.1.2 Loss function

During training, we utilized the Cross-entropy loss and the Dice loss as follows:


(1) $$L_{\mathrm{total}}=L_{\mathrm{dice}}+L_{ce}$$

where the Cross-entropy loss *L_ce_* is defined as follows:


(2) $$L_{ce}=-\frac{1}{N}\underset{i}{\sum }\overset{K}{\underset{k}{\sum }}v_{ik}\log\left(u_{ik}\right)$$

Dice loss was first introduced in (23) to solve the imbalance between positive and negative samples. Dice loss is different from Cross-entropy loss: it helps minimize segmentation error and obtain more stable segmentation performance (36). The Dice loss equation is as follows:


(3) $$L_{\mathrm{dice}}=-\frac{2}{\left|K\right|}\underset{k\in K}{\sum }\frac{\sum_{i\in i}u_{i}^{k}v_{i}^{k}}{\sum_{i\in I}\;u_{i}^{k}+\sum_{i\in i}v_{i}^{k}}$$

In Eqs. (2) and (3), *u* is the model’s predictive probability, *v* is the ground truth one-hot code, *K* is the number of classes, and *I* is the representation vector of the image. Empirically, Cross-entropy loss makes the model focus on the global representation, i.e., each pixel of the image, while Dice loss pays more attention to the positive region, so *L_total_* takes advantage of both global and local information.

##### 3.1.1.3 Inference

Image segmentation inference was then performed on patches of MRI images. An image was divided into patches with an overlap of *size/2* pixels, where *size* is the size of the stride. Due to the lack of neighbor information, the segmentation accuracy of the edges of each patch will be relatively lower, so when fusing the segmentation result for pixels along the edges, we decreased the weight of edge pixels while increasing the weight of pixels close to the center.

##### 3.1.1.4 Post-processing

After obtaining a segmentation result, we found the largest connected contour and, in the meantime, neglected other smaller ones. This post-processing step can effectively reduce the occurrence of false positives.

#### 3.1.2 Two-step lesion segmentation

In order to reduce the probability of false positives, we utilized a two-step lesion segmentation method to extract the breast cancer lesion region. As shown in Figure 1, given a DCE-MRI sample for breast cancer diagnosis, we first segmented the mammary gland region, based on which we then segmented the breast cancer lesion region.

##### 3.1.2.1 Mammary gland segmentation

The pre-processing described in Section 3.1.1 was applied to the original DCE-MRI samples, and the backbone network, i.e., nnUNet, was utilized to implement the first step “mammary gland segmentation” task.

##### 3.1.2.2 Breast cancer lesion segmentation

After getting the result from the first segmentation step, we continued to segment the breast cancer lesion region. The mammary gland region was pre-processed only by Z-score normalization and is fed to the second segmentation step. We used the same backbone network to implement the “breast cancer lesion segmentation” task.

The details of the training are explained in Section 4, and the performance of our proposed two-step lesion segmentation method is also shown in the following section.

#### 3.1.3 Domain adaptation

As our dataset was collected from two different centers, there will inevitably be a model transfer issue when training on samples from one center and testing on another. This is a common issue in medical image analysis because different medical imaging devices with different imaging protocols, methods, and different operators produce MRI images that vary in resolution, quality, etc.; therefore, many methods have been proposed to mitigate this issue (13, 37, 38). With respect to the specific differences between DCE-MRI samples, we designed a domain adaptation method, i.e., simple histogram matching (13), to improve the transferability of the model. Another advantage of histogram matching is that it only requires the gray-level distribution of the DCE-MRI images; thus, it does not reveal any personal information about the patient.

##### 3.1.3.1 Histogram matching

At this point, we applied a simple histogram matching method (13) to augment training samples in order to improve transferability. More specifically, we introduced the gray-level distribution to augment training samples, each of which was augmented by matching the gray-level histogram computed with samples from other centers. The histogram matching is implemented as follows:


(4) $$S_{k}=\frac{L-1}{M*N}\overset{k}{\underset{j=0}{\sum }}n_{j}*k=0,1,2\ldots ,L-1$$

where *L* is the maximum gray-level value of the target histogram, *M* and *N* are the width and height of the image, and *n_j_* is the gray-level value of pixel *j*.

As for the segmentation task, we first implemented the mammary gland segmentation without histogram matching. We then computed a gray-level histogram for each sample in the test dataset (in our experiment, samples from Henan Renmin Hospital are used as the test dataset) and then applied histogram matching to each sample in the training dataset (in our experiment, samples from Guangdong Provincial People’s Hospital are used as the training dataset) with a randomly selected gray-level histogram from the test dataset. After the training dataset was augmented, it was fed to nnUNet for breast cancer lesion segmentation training.

### 3.2 Pathologic complete response prediction

Among all treatments for breast cancer, NAC is emerging as a new and effective method. As introduced in Section 1, utilizing imaging examination as a non-invasive method, together with four types of molecular typing data commonly used in breast cancer treatment, we proposed a multi-modal fusion model to predict whether axillary lymph nodes could achieve pCR after patients receive NAC.

#### 3.2.1 Multi-modal fusion

Although one can use the MRI data of the breast cancer lesion to directly predict the probability of pCR after neoadjuvant therapy, it has been proven that immunohistochemical detection can also help in breast cancer prognosis (14). Thus, we propose to utilize common types of molecular typing data extracted by immunohistochemical detection of breast cancer. More specifically, we chose the following four common types of molecular typing data in breast cancer treatment: Human Epidermal Growth Factor Receptor 2 (HER2), Estrogen Receptor (ER), Progesterone Receptor (PR), and Ki-67.

HER2 protein is negative in normal breast tissue, and the amplification of HER2 is highly related to the growth, proliferation, transfer, and invasion of tumor cells; thus, it can be treated as one of the prognostic indicators of clinical treatment monitoring. ER and PR are nuclear hormone receptors; the expression of ER/PR indicates that tumor cells retain the characteristics of hormone-dependent growth and is significant in the prognosis judgment of breast cancer. Ki-67 is a monoclonal antibody; high expression of Ki-67 indicates a poor prognosis.

In our work, we used the above four types of molecular typing data, together with a DCE-MRI image of breast cancer lesions, to train the multi-modal (i.e., text and image) fusion model to predict pCR.

#### 3.2.2 Network structure

**Figure 3:**
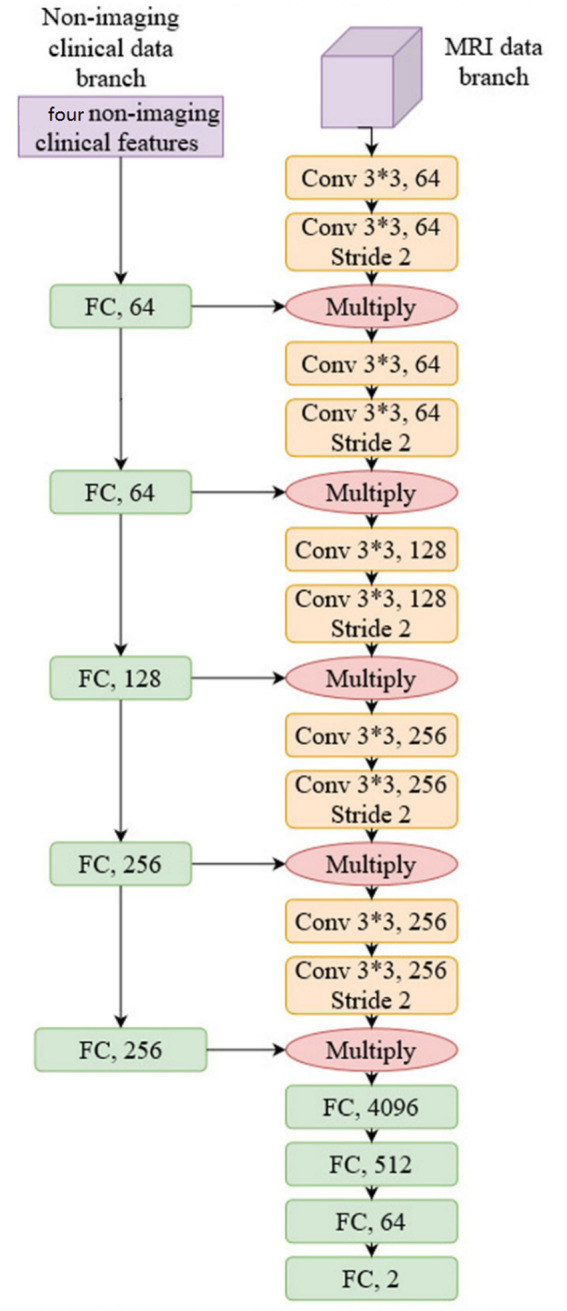
The network structure of our multi-model fusion model for pCR prediction.

We used conventional ResNet (18) as the backbone network to construct the prediction model as a common practice; more specifically, ResNet34 was selected, and we justify this choice in Section 4. The network structure is shown in Figure 3:

As shown in Figure 3, non-imaging features and MRI data were processed by two separate network branches. The molecular typing data was processed by five Fully Connected (FC) layers, while the MRI image was processed by five convolutional network layers. It should be noted that the output of each FC layer in the non-imaging clinical data branch is fused with the output of each CNN layer in the MRI data branch by multiplication. This structure balances the weight of non-imaging data and MRI data to compute the model representation and makes the model utilize both molecular typing data and a DCE-MRI image of a breast cancer lesion to predict pCR. The proposed network is trained by the conventional Cross-entropy loss function.

## 4 Experiments and analysis

### 4.1 Experiment setting

#### 4.1.1 Dataset

In our work, we used DCE-MRI data for breast cancer lesion segmentation and pCR prediction. DCE-MRI can provide a high-quality image for soft tissues with better quality of blood flow around the lesion region, which facilitates higher accuracy and earlier detection in breast cancer diagnosis. Therefore, DCE-MRI is the most widely adopted imaging method in breast cancer diagnosis and treatment.

**Figure 4:**
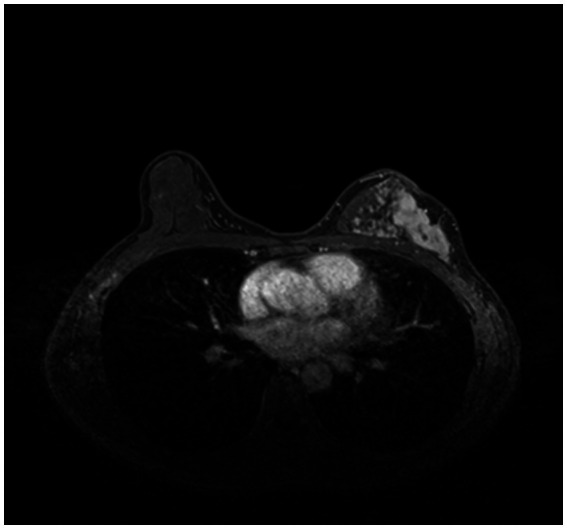
An example of the 361 labeled DCE-MRI samples in our dataset.

In order to train and test the proposed method, we collected 361 breast cancer samples from two hospitals: 246 samples from Guangdong Provincial People’s Hospital and 115 samples from Henan Renmin Hospital. Each DCE-MRI image was labeled and verified by professionals. A labeled DCE-MRI sample is shown in Figure 4. We also collected the four types of molecular typing data commonly used in breast cancer treatment: HER2, ER, PR, and Ki-67, for each of the 361 samples.

#### 4.1.2 Network training setup

For breast cancer lesion segmentation, the network was trained by Adam optimizer (39) with a learning rate of 3e-4 for 1,000 epochs. The learning rate was decayed by 5 if the decrease in the average training loss over 30 epochs was less than 5e-3. The model convergence criteria are: the decrease in the average training loss over 60 epochs must be less than 5e-3, or the learning rate must be less than 1e-6. For pCR prediction, after acquiring the segmentation result, the breast cancer lesion images were resampled to a size of 128*128*128. The initial learning rate was set to 1e-4, and the network was trained for 200 epochs.

### 4.2 Ablation studies

#### 4.2.1 Effect of histogram matching

**Figure 5:**
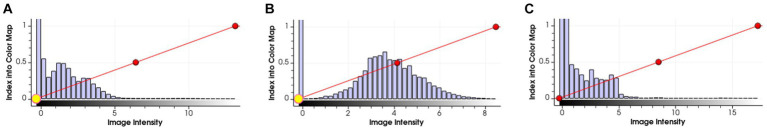
Example of gray-level histogram matching. **(A)** is a sample from Guangdong Provincial People’s Hospital; **(B)** is a randomly picked sample from Henan Renmin Hospital; and **(C)** is the result of applying histogram matching to **(A)**.

As introduced in Section 3.1.3, we used a simple histogram matching method to augment the training dataset in order to improve the transferability of the model. Here, we conducted an ablation study to show the effect of this domain adaptation method. As shown in Figure 5, for an MRI image (a) from the training dataset (i.e., samples from Guangdong Provincial People’s Hospital), we randomly picked a target image (b) from Henan Renmin Hospital and applied gray-level histogram matching to the original training image so as to obtain an augmented sample (c).

**Figure 6:**
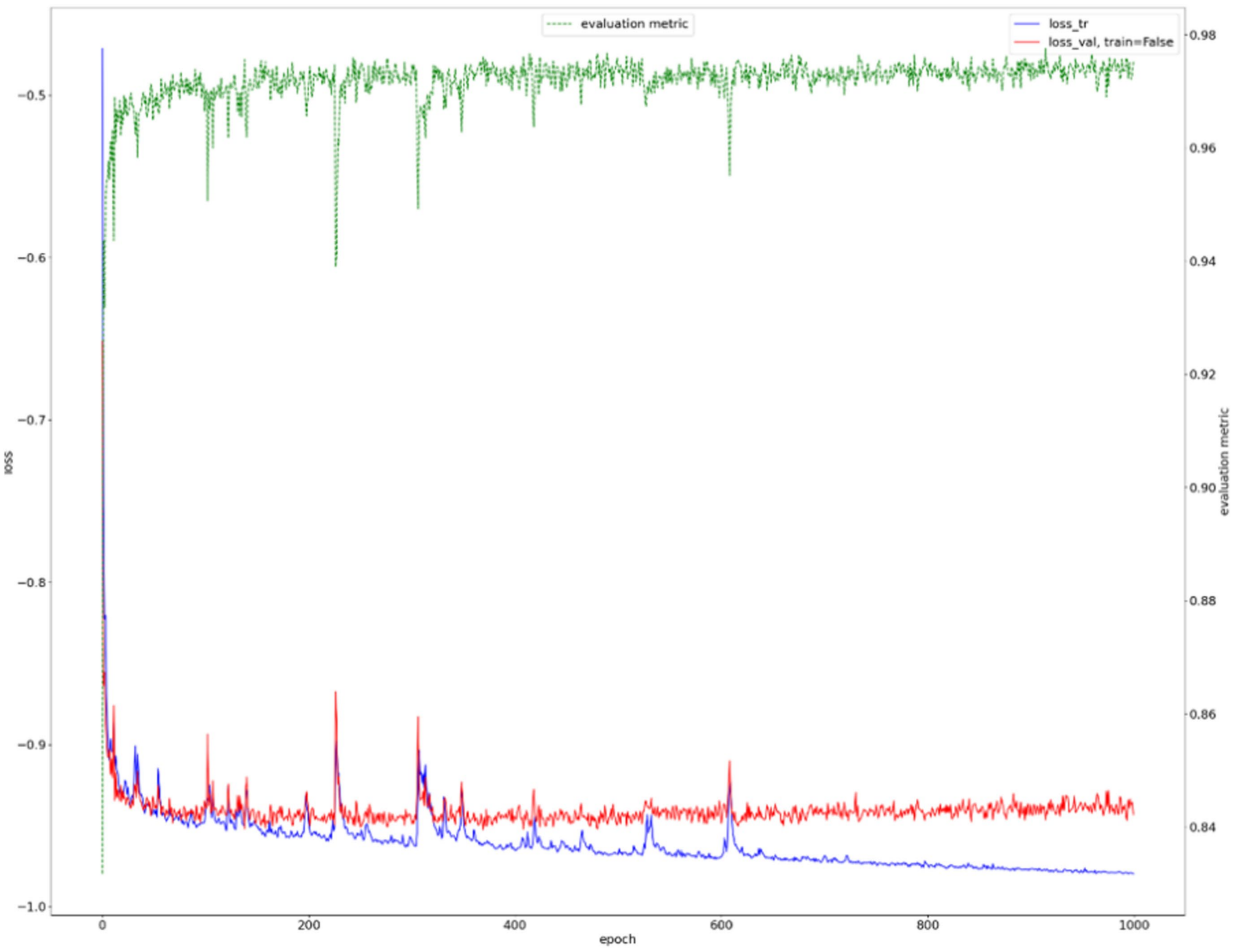
Training curve of mammary gland segmentation.

We show the results of the proposed segmentation model with and without domain adaptation in Table 1. It is apparent that after applying the proposed domain adaptation method, i.e., histogram matching, the segmentation IoU increased by 7%. We also note that the training curve oscillates more than it does without histogram matching, as shown in Figure 6. This is because the gray-level distribution of the target dataset is introduced into the training samples.

**Table 1:** Performance of the breast cancer lesion region segmentation task with and without domain adaptation.

Task	Domain Adaptation	Dice	IoU
Breast cancer lesion region segmentation	No	0.78	0.65
	Yes	0.83	0.72

#### 4.2.2 ResNet depth

Normally, a deeper network implies stronger modeling ability; however, this is not always true in medical image processing models because of the higher risk of overfitting. We conducted an ablation study to show how the depth of the ResNet affects the model’s performance. We trained the pCR prediction model with conventional ResNet18, ResNet34, and ResNet50, respectively, and the result is shown in Table 2. We can see that the performance does not change much between models with different ResNet depths. Based on this, we chose ResNet43 in the experiments that follow.

**Table 2:** Result of pCR prediction with ResNet of different depths.

Task	ResNet depth	Accuracy	AUC
pCR prediction	ResNet18	0.70	0.68
	ResNet34	0.72	0.69
	ResNet50	0.71	0.69

#### 4.2.3 Effect of the surrounding mammary gland

**Figure 7:**
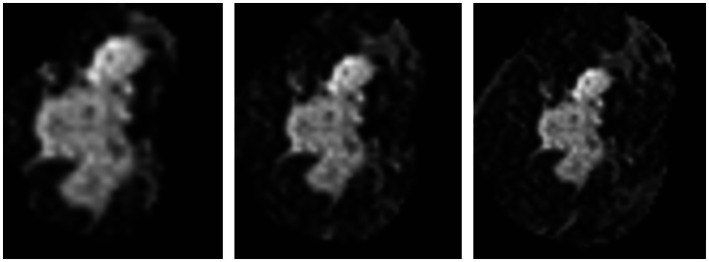
Example of expanding a segmented lesion region by 5, 10 and 15 pixels, respectively, from left to right.

The performance of the prediction model is directly affected by the correlation between the input data and the prediction target. As for pCR prediction tasks, as pointed out in (40), the mammary gland provides certain information when determining preoperative lymph node metastasis in breast cancer. Also, as pointed out by professionals, the MRI data of the mammary gland may contain abnormal information that may be related to patient prognosis. Thus, we conducted an ablation to test the influence of the surrounding mammary gland in predicting pCR. After acquiring the segmentation result of the breast cancer lesion, we expanded the periphery by using an expansion algorithm with kernels of three sizes, i.e., 5 pixels, 10 pixels, and 15 pixels. An example is shown in Figure 7. We used a circular expansion kernel in order to maintain the original shape of the segmented lesion. Then the expanded DCE-MRI data of the lesion region was used to train the proposed pCR prediction model, and the result is shown in Table 3. It is quite obvious that the surrounding gland information does not help at all in pCR prediction, so we used the segmented lesion region directly for pCR prediction in the following experiments.

**Table 3:** Result of pCR prediction with different expanding kernel sizes.

Task	Kernel size (pixel)	Accuracy	AUC
pCR prediction	-	0.72	0.69
	5	0.42	0.60
	10	0.57	0.77
	15	0.42	0.76

#### 4.2.4 Effects of multi-modal fusion

We also conducted an ablation study to show the effect of multi-modal fusion. We present the performance of pCR prediction with only DCE-MRI data of the segmented lesion and with both DCE-MRI data and four common types of molecular typing data (i.e., multi-modal fusion) in Table 4. It is noted that the multi-modal fusion model provides a 13% increase in accuracy, which proves that the proposed model is effective.

**Table 4:** Result of pCR prediction with DCE-MRI data only and with multi-modal fusion.

Task	Model	Accuracy	AUC
pCR prediction	ResNet34	0.72	0.69
	Multi-modal fusion	0.85	0.81

### 4.3 Experiment results

In this section, we present the results of the two-step lesion segmentation and pCR prediction. It should be noted that the experiments were conducted according to the method introduced in Section 3, and as explained in Section 4.2, the experiments were performed with domain adaptation, with ResNet34, without the surrounding mammary gland data of the lesion, and with multi-modal fusion.

#### 4.3.1 Two-step lesion segmentation

##### 4.3.1.1 Mammary gland segmentation.

**Figure 8:**
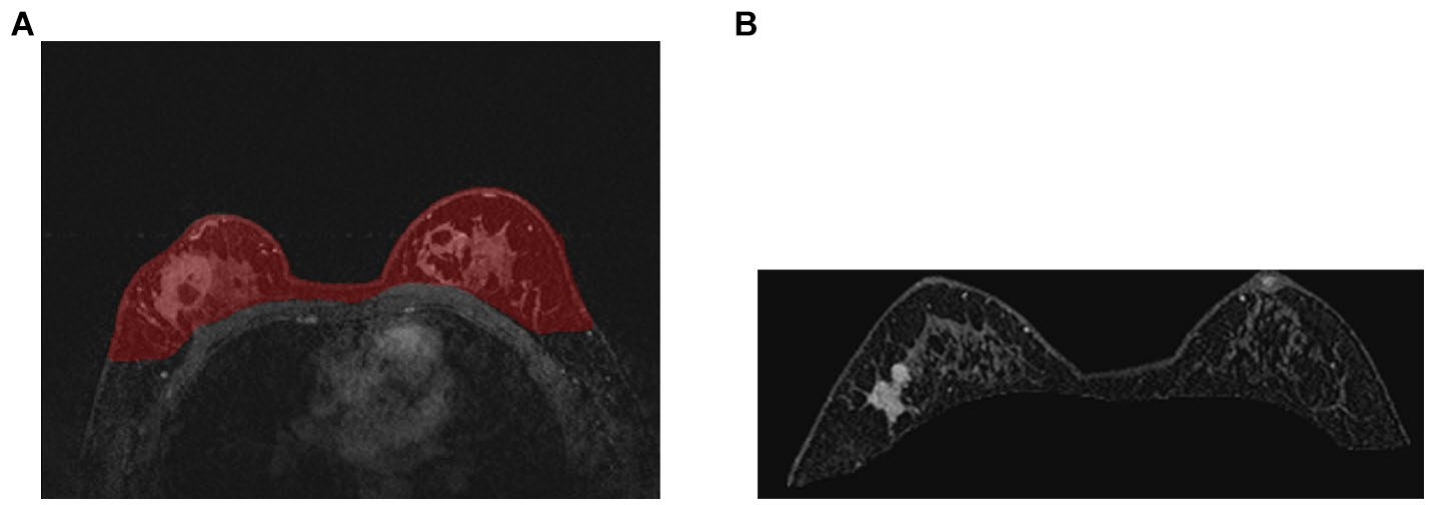
Examples of segmented mammary gland. **(A)** shows the mammary gland region in red, **(B)** shows a segmented mammary gland.

The training curve is shown in Figure 6 and examples of segmented mammary glands are shown in Figure 8.

The performance of the proposed method for mammary gland segmentation is shown in Table 5. We achieved 93% IoU in the first segmentation task.

**Table 5:** Performance of the two-step lesion segmentation task.

Task	Dice	IoU	HD95
Mammary gland segmentation	0.96 ± 0.01	0.93 ± 0.02	3.73 ± 2.02
Breast cancer lesion segmentation	0.83	0.72	-

##### 4.3.1.2 Breast cancer lesion segmentation.

**Figure 9:**
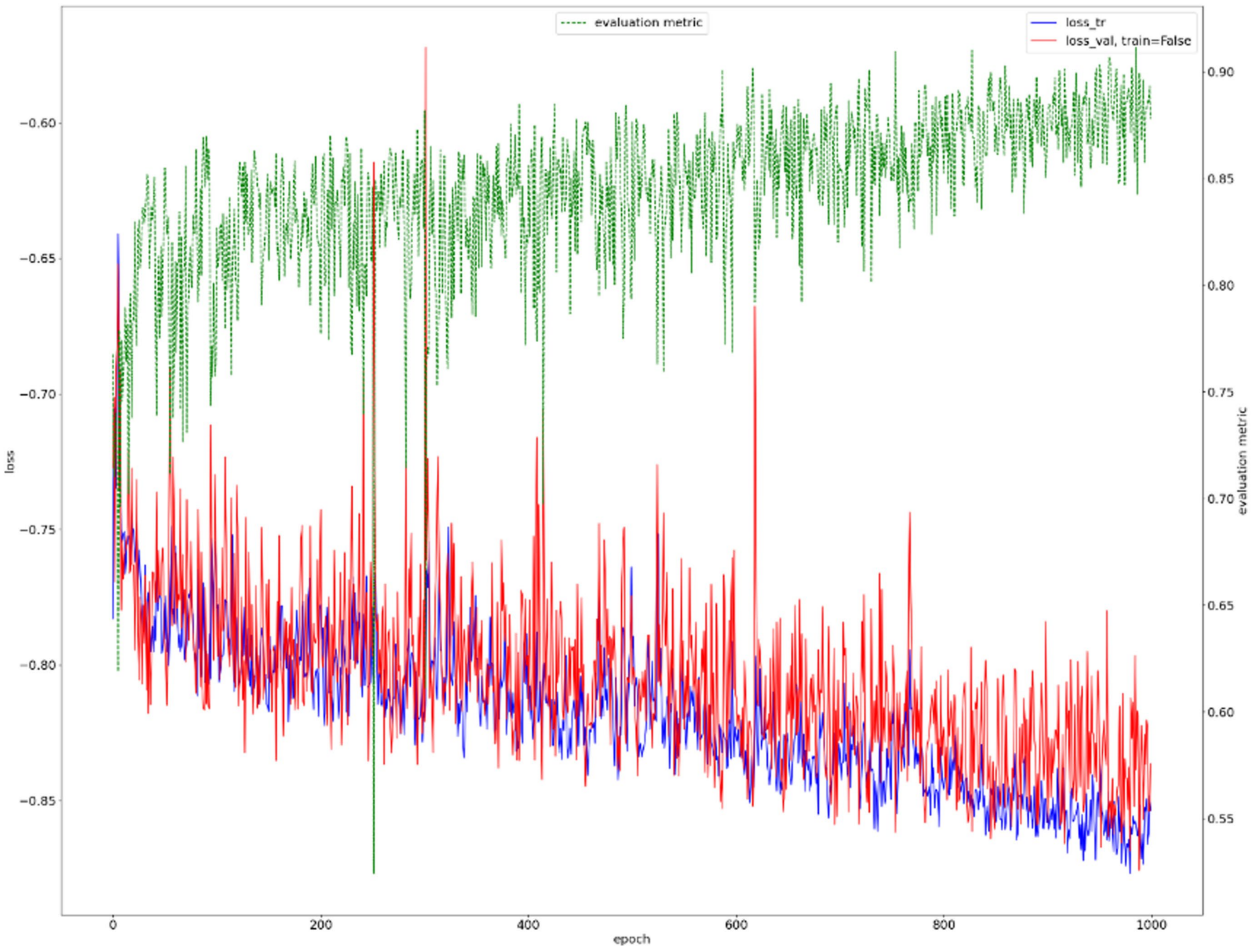
Training curve of breast cancer lesion segmentation.

Results from the first segmentation step, for example, Figure 8B, were used as input for the second segmentation step. The training curve is shown in Figure 9, and the performance of the proposed method for breast cancer lesion segmentation is shown in Table 5.

#### 4.3.2 Pathologic complete response prediction

We used a multi-modal fusion model to predict whether axillary lymph nodes could achieve pCR after patients receive neoadjuvant therapy. DCE-MRI data of the segmented breast cancer lesion region and four types of molecular typing data commonly used in breast cancer treatment (i.e., HER2, ER, PR, and Ki-67) were utilized as input to the proposed multi-modal fusion model. The performance of pCR prediction by the proposed model is shown in Table 4. The multi-modal fusion model achieved an accuracy of 85%, which is significantly high for pCR prediction of breast cancer with only non-invasive methods.

In addition, we performed McNemar’s test of the two pCR prediction methods, one with DCE-MRI data only and the other with multi-modal fusion. We also performed a Chi-square goodness-of-fit test between each of the two methods and the ground truth. We randomly selected 200 samples to test each of the two methods, and the statistics of the pCR prediction results are shown in Tables 6, 7, respectively. The McNemar’s test showed that there exists a statistical difference between the two pCR prediction methods, while the Chi-square goodness-of-fit test revealed that the pCR prediction result by the multi-modal fusion method is more consistent with the ground truth distribution.

**Table 6:** Result of McNemar’s test of two pCR prediction methods.

Prediction with DCE-MRI	Prediction with Multi-modal		Total	*χ* ^2^	*p*
	pCR	non-pCR			
pCR	63	1	64	9.31	0.0023
non-pCR	12	124	136		
Total	75	125	200		

**Table 7:** Result of the Chi-square goodness-of-fit test between each of the two pCR prediction methods and the ground truth, respectively.

	pCR	non-pCR	*χ* ^2^	*p*
Prediction with DCE-MRI	64	136	7.43	0.0064
Prediction with Multi-modal	75	125	1.32	0.2509
Ground truth	83	117		

## 5 Conclusion

In this paper, we presented a two-step lesion segmentation method to extract breast cancer lesion regions from DCE-MRI images, and in this process, we applied a simple histogram matching method to improve the transferability of the model. Then, we proposed a multi-modal (i.e., segmented DCE-MRI image and molecular typing data) fusion model to predict the probability of axillary lymph nodes achieving pCR after patients receive NAC. We collected 361 breast cancer samples from two hospitals to train and test the proposed segmentation method and the multi-modal fusion model. We demonstrated that our method achieves 93 and 72% IoU in mammary gland segmentation and breast cancer lesion segmentation tasks, respectively. We also showed that our multi-modal fusion model is effective and reaches 85% accuracy in pCR prediction using only data collected in a non-invasive manner. Although the IoU of breast cancer lesion segmentation is not very high (72%), it was used in the multi-modal fusion model and reached 85% accuracy in pCR prediction. This suggests that the presented method can be used for the prediction of treatment responses in breast cancer.

### 5.1 Limitations

The 361 breast cancer samples we collected for this study only include patients with solid tumors; therefore, this study focuses on lesion region segmentation of solid tumors and cannot be directly applied to other types of lesions, e.g., non-mass lesions or different breast parenchyma compositions. If the proposed method is to be used in other cases, it needs to be re-trained with enough specific data samples. Additionally, the proposed pCR prediction method requires a two-step process where we need to segment the breast cancer lesion from the DCE-MRI image, only then can we perform the final pCR prediction with the multi-model fusion model.

## Data availability statement

The data analyzed in this study is subject to the following licenses/restrictions: The image and clinical medical data used to support the findings of this study are restricted by the Ethics Board of Guangdong Provincial People’s Hospital in order to protect patient privacy. Data are available from Gang Fang, gangf@gzhu.edu.cn for researchers who meet the criteria for access to confidential data. Requests to access these datasets should be directed to Gang Fang, gangf@gzhu.edu.cn.

## Ethics statement

The studies involving human participants were reviewed and approved by the Ethics Board of Guangdong Provincial People’s Hospital and Ethics Board of Henan Renmin Hospital. Written informed consent to participate in this study was provided by the participants.

## Author contributions

GF designed and supervised the study. YL carried out the whole study. ZC assisted the whole study, conducted extra experiments. JC and ZS wrote the code for the study. All authors contributed to the article and approved the submitted version.
